# Clinical characteristics and therapeutic outcomes of nosocomial super-infection in adult bacterial meningitis

**DOI:** 10.1186/1471-2334-11-133

**Published:** 2011-05-18

**Authors:** Chi-Ren Huang, Shu-Fang Chen, Cheng-Hsien Lu, Yao-Chung Chuang, Nai-Wen Tsai, Chiung-Chih Chang, Hung-Chen Wang, Chun-Chih Chien, Wen-Neng Chang

**Affiliations:** 1 Department of Neurology, Kaohsiung Chang Gung Memorial Hospital and Chang Gung University College of Medicine, Kaohsiung, Taiwan; 2 Department of Biological Science, National Sun Yat-Sen University, Kaohsiung, Taiwan; 3 Neurosurgery, Kaohsiung Chang Gung Memorial Hospital and Chang Gung University College of Medicine, Kaohsiung, Taiwan; 4 Diagnostic Pathology, Kaohsiung Chang Gung Memorial Hospital and Chang Gung University College of Medicine, Kaohsiung, Taiwan

## Abstract

**Background:**

Super-infection in adult bacterial meningitis (ABM) is a condition wherein the cerebrospinal fluid (CSF) grows new pathogen(s) during the therapeutic course of meningitis. It is an uncommon but clinically important condition rarely examined in literature.

**Methods:**

Twenty-seven episodes of super-infection states in 21 ABM patients collected in a 9.5-year study period (January 2001 to June 2010) were evaluated. The clinical characteristics, implicated pathogens, results of antimicrobial susceptibility tests, and therapeutic outcomes were analyzed.

**Results:**

Twenty-one patients (13 men, 8 women) aged 25-73 years (median, 45 years) had post-neurosurgical state as the preceding event and nosocomial infection. The post-neurosurgical states included spontaneous intracranial hemorrhage (ICH) with craniectomy or craniotomy with extra-ventricular drainage (EVD) or ventriculo-peritoneal shunt (VPS) in 10 patients, traumatic ICH with craniectomy or craniotomy with EVD or VPS in 6 patients, hydrocephalus s/p VPS in 2 patients, and one patient each with cerebral infarct s/p craniectomy with EVD, meningeal metastasis s/p Omaya implant, and head injury. All 21 patients had EVD and/or VP shunt and/or Omaya implant during the whole course of ABM. Recurrent fever was the most common presentation and the implicated bacterial pathogens were protean, many of which were antibiotic resistant. Most patients required adjustment of antibiotics after the pathogens were identified but even with antimicrobial therapy, 33.3% (7/21) died. Morbidity was also high among survivors.

**Conclusions:**

Super-infection in ABM is usually seen in patients with preceding neurosurgical event, especially insertion of an external drainage device. Repeat CSF culture is mandatory for diagnostic confirmation because most of the implicated bacterial strains are non-susceptible to common antibiotics used. Unusual pathogens like anaerobic bacteria and fungi may also appear. Despite antimicrobial therapy, prognosis remains poor.

## Background

Super-infection in adult bacterial meningitis (ABM) is a condition in which the cerebrospinal fluid (CSF) grows new pathogen(s) during the therapeutic course of meningitis [1,2]. This uncommon situation is rarely examined solely in overview studies of ABM [3,4]. In ABM management, early and appropriate antibiotic use is an important strategy for better outcomes [5-7]. However, super-infection may cause a dilemma in antibiotic choice because starting a new antimicrobial regimen for this complicated condition during the therapeutic course of ABM is a difficult decision. To better delineate the clinical characteristics of super-infection in ABM, this study analyzed the clinical characteristics and therapeutic outcomes of ABM patients with super-infection.

## Methods

The medical and microbiological records for CSF of all adult patients with bacterial meningitis admitted to Chang Gung Memorial Hospital (CGMH)-Kaohsiung, a 2482-bed acute-care teaching hospital and the largest tertiary care center in southern Taiwan, from January 2001 to June 2010 (9.5 years) were retrospectively reviewed. The hospital's Ethics Committee approved the study (IRB 99-1897C).

Diagnosis of ABM was based on: i) age ≥17 years; ii) positive CSF culture in patients with clinical presentation of acute meningitis (i.e. fever, altered consciousness, or seizure); and iii) at least one of the following CSF parameters: leukocyte count >0.25 × 10^9^/L with predominant polymorphonuclear cells; CSF lactate concentration >3.5 mmol/L; glucose ratio (CSF glucose/serum glucose) <0.4; or CSF glucose concentration <2.5 mmol/L if no simultaneous blood glucose was determined [3,4]. "Super-infection" in ABM was defined as a condition wherein CSF grew new pathogen(s) during the therapeutic course of an existing bacterial meningitis [1,2].

For this study, ABM was classified as either nosocomial or community-acquired. Nosocomial meningitis was defined as positive bacterial infection not present on hospital admission, clinical evidence of infection no sooner than 48 hours after admission, or clinical evidence of meningitis within a short period of time, i.e. within one month after discharge from the hospital where the patient received an invasive procedure, especially a neurosurgical one. Otherwise, the patient was considered as having "community-acquired" infection.

Patients who developed meningitis related to head trauma with skull fractures or neurosurgical procedures were classified as "post-neurosurgical" meningitis. Otherwise, patients who demonstrated no clear distinctive disease characteristics and who had not undergone any invasive procedures were classified as "spontaneous" meningitis. "Mixed-infection" was defined as at least two bacterial organisms isolated concomitantly from CSF culture [4,8].

During the study period, intravenous administration of vancomycin with either a 3^rd ^- or 4^th^- generation cephalosporin (e.g. ceftriaxone, ceftazidime, or cefepime) were the initial empiric antibiotics used to treat adult patients with clinical evidence of bacterial meningitis. Further antibiotic adjustment was guided by results of pathogen identification and antibiotic susceptibility tests. Antibiotic susceptibility of the isolated pathogen was tested using the Kirby-Bauer diffusion method (BBL, Muller-Hinton II agars; Becton Dickinson Microbiology Systems, Cockeysville, MD) while bacterial susceptibility to antimicrobial agents was determined according to criteria of the National Committee for Clinical Laboratory Standards (CLSI). The therapeutic results were evaluated by using Glasgow Outcome Scale (GOS) [9]: score 1 = death, score 2 = persistent vegetative state, score 3 = severe disability, score 4 = moderate disability, score 5 = good recovery. Scores of 1-3 were classified as "poor prognosis" while scores of 4 and 5 were "good prognosis".

For comparative analysis, the patients were divided into fatal and non-fatal groups. Data such as sex, type of infection, underlying conditions, and clinical manifestations between these two patient groups were analyzed by Fisher's exact test. Age between the two groups was analyzed by Mann-Whitney U test.

## Results

During the study period, 21 ABM patients aged 25-73 years (median 45 years), including 13 men and 8 women, had super-infection. Their basic clinical and laboratory data are listed in Tables 1 and 2. All had post-neurosurgical state as the preceding event and had contracted the infection nosocomially. The post-neurosurgical states included spontaneous intracranial hemorrhage (ICH) with craniectomy or craniotomy with extra-ventricular drainage (EVD) or ventriculo-peritoneal shunt (VPS) in 10 patients (Cases 2-4, 9-11, 14, 17, 20, 21), traumatic ICH with craniectomy or craniotomy with EVD or VPS in 6 patients (Cases 5, 6, 12, 13, 16, 18), hydrocephalus s/p VPS in 2 patients (Cases 7, 15), cerebral infarct s/p craniectomy with EVD in Case 1, meningeal metastasis s/p Omaya implant in Case 8, and head injury in Case 19. All 21 patients had undergone EVD and/or VPS and/or Omaya implant during the whole clinical course of ABM. The underlying medical conditions included diabetes mellitus in 3 patients (Cases 3, 10, 20), lung cancer in Case 8, brain tumor in Case 11, and liver cirrhosis in Case 21.

**Table 1:** Clinical data and implicated pathogens in the super-infections of the study patients with adult bacterial meningitis

Case	Gender	Age (years)	Underlying condition	Initial pathogen(s)	Antibiotics	Management	Interval* (days)	New presentation	New pathogen(s)	Antibiotics	Management	Survived
More than 7 days interval*												

	Gram (+) → G (-) or G (-) → G (+)											

1	M	55	Infarct, craniectomy, EVD	*Staphylococcus aureus*^§^	LZD+EPM	Removed EVD → new EVDs	61	Fever, leukocytosis, CSF pleocytosis	ESBL-*Escherichia coli*	MEP	Removed EVD	No
2	F	48	SICH, craniectomy, EVD	*Staphylococcus aureus*^§^	VA+CAZ	Removed EVD → craniotomy, new EVDs	34	Fever, hydrocephalus	*Stenotrophomonas maltophilia*^*#*^	MOX+TMP+ TIG+ ATM	Removed EVD → New EVD → removed EVD → VP shunt	Yes
3	F	55	DM, SICH, craniotomy, VPS	*Staphylococcus aureus*^§^	VA	Removed VPS, debridement → new EVD	11	Fever, CSF pleocytosis	*Acinetobacter baumannii*	LZD+MEP	New EVD → ventriculo-pleural shunt	Yes
4	F	41	SICH, craniectomy, EVD	*Staphylococcus epidermidis*^§^	VA	Craniotomy, EVD → VPS	47	No	*Enterobacter cloacae *+ *Pseudomonas aeruginosa*	CAZ	Removed VPS	No
5	M	73	TICH, craniectomy, EVD (removed)	Coagulase-negative staphylococci^§^	VA	New EVD	9	Fever, seizure, altered consciousness	*Enterobacter cloacae*	ROC	Removed EVD → VP shunt	Yes
6	M	46	TICH, craniectomy, VPS	Coagulase-negative staphylococci^§^	VA	Externalisation VPS → removed VPS → new EVDs	46	Fever	Coagulase-negative staphylococci^§ ^+ *Acinetobacter baumannii*	MEP	Removed EVD → new EVDs → VP shunt	Yes
7	M	34	Hydrocephalus, VPS	*Staphylococcus epidermidis *^§^	VA	Removed VPS, New EVDs	28	Fever, CSF pleocytosis	*Acinetobacter spp*	CEF	Removed EVD → New EVD → removed → VPS	Yes
8	F	45	Lung cancer, meningeal metastasis, Omaya implant, infection	Coagulase-negative staphylococci^§^	VA	Debridement, removed Omaya implant	42	Fever	*Acinetobacter spp*	CAZ		Yes
9	F	48	Brain tumor, SICH, craniectomy	*Pseudomonas aeruginosa*	CEF	New EVD	14	No	*Enterococcus spp*	AMP+CEF	New EVD → Removed EVD	Yes
10	M	67	DM, SICH, VPS, craniotomy, EVD	*Acinetobacter baumannii*	MEP	Removed VPS, new EVDs	41	Fever	*Staphylococcus aureus*^§^	VA	Removed EVD	No

	Gram (-) → G (-)											

11A	M	45	Brain tumor, SICH, craniectomy, VPS	*Serratia marcescens*^@^	MEP	Removed VPS, new EVD	29	Fever, reduced CSF glucose	*Stenotrophomonas maltophilia*	MOX+TMP	Removed EVD → new EVD	Treated
11B							90	No	*Candida glabrata*	FLU+AMB		Treated
11C							48	Fever	*Stenotrophomonas maltophilia*^*#*^	TMP+TIG		No
12	M	25	TICH, craniectomy, EVD	*Pseudomonas aeruginosa*	CAZ	New EVDs	37	No	*Pseudomonas aeruginosa *+ ESBL-*Proteus mirabilis*	MEP	Removed EVD	Yes
13	M	25	TICH, craniotomy, VPS	*Pseudomonas putida*	CAZ	Removed VPS, new EVD	23	Leukocytosis	*Acinetobacter spp*^@^	MEP+SBM	Removed EVD → New EVDs → VPS	Yes
14	M	64	SICH, VPS	*Serratia marcescens*	VA + MEP	Removed VPS → new EVD	8	Fever, altered consciousness, hydrocephalus	*Acinetobacter baumannii*^@^	MEP+SBM+ TIG+CST	Removed EVD → new EVDs → VP shunt	Yes
15A	F	71	Old meningitis, hydrocephalus, VPS	*Morganella morganii*	CAZ	Externalization VPS→ removed → new VPS	47	Fever	*Proteus mirabilis*^@^	MEP	Removed VPS	Treated
15B							40	Fever	Glucose non-fermenting bacilli^@^	MEP+SBM	Scalp debridement → VPS	Yes
16	M	31	TICH, craniotomy, EVD (removed)	*Stenotrophomonas maltophilia*	CAZ	New EVDs	60	Fever	*Stenotrophomonas maltophilia *+ *Pseudomonas aeruginosa*	MEP+CIP	New EVDs → VPS → removed VPS → Omaya implant	Yes

	Others											

17	F	58	SICH, EVD	*Staphylococcus epidermidis*^§^+ *Acinetobacter lwoffii*	VA+ROC+ CM	Removed EVD, new EVD	28	No	*Staphylococcus epidermidis*^§^+ *Pseudomonas aeruginosa*	CAZ	Removed EVD → New EVD → VPS	Yes
18A	M	48	TICH, craniectomy, EVD (removed)	*Escherichia coli*	MEP	New EVDs → craniotomy	36	Scalp wound infection	*Candida tropicalis*	FLU	removed EVD, debridement	Treated
18B							9	No	*Escherichia coli*+ *Acinetobacter bauamnnii*+ *Enterococcus faecalis*	AMP	Debridement → new EVD	Treated
18C							11	Fever	*Staphylococcus aureus*^§^+ *Acinetobacter baumannii*	VA+IPM	Removed EVD, new EVD	Treated
18D							14	Fever	*Stenotrophomonas maltophilia*	CIP	Removed EVD, new EVD → removed	Yes
19	M	58	Head injury	Viridan streptococci	VA+ROC		8	Hydrocephalus, ventriculitis, CSF pleocytosis	*Prevotella*+ *Veilonella*+ *Fusobacterium*	VA+ROC+ MET	New EVD	No

Less than 7 days interval												

20	F	64	DM, VHD, SICH, craniectomy, EVD	*Escherichia coli*+ *Enterococcus faecalis*	VA+CAZ	Removed EVD, New EVD	3	Fever, leukocytosis, CSF pleocytosis	*Enterobacter cloacae*^*#*^^@^	VA+CAZ	Removed EVD → VPS	No
21	M	49	Liver cirrhosis, SICH, craniectomy, EVD (removed)	Coagulase-negative staphylococci^§^	VA	New EVD	5	No	*Enterobacter aerogenes*	CEF	New EVDs	No

^§^Oxacillin-resistant; ^*#*^change antibiogram during treatment; ^@ ^cephalosporin-resistant; *the time interval from the finding of previous pathogen(s) to the detection of the following new pathogen(s).

Abbreviations: M, male; F, female; EVD, external ventricular drainage; SICH, spontaneous intra-cranial hemorrhage; DM, diabetes mellitus; VPS, ventriculo-peritoneal shunt; TICH, traumatic intra-cranial hemorrhage; ESBL, extended spectrum β-lactamase; LZD, linezolid; EPM, entrapenem; VA, vancomycin; CAZ, ceftazidime; CEF, cefepime; MEP, meropenem; ROC, ceftraixone; CM, chloroamphenicol; CSF, cerebrospinal fluid; MOX, moxifloxacin; TMP, trimethoprim-sulfamethoxazole; FLU, fluconazole; AMB, amphotericin B; TIG, tigecycline; SBM, sulbactam; ATM, aztreonam; CST, colistin; AMP, ampicillin; MET, metronidazole

There were 27 episodes of super-infection in the 21 ABM patients. The initial and subsequently implicated pathogens are listed in Table 1. Eight patients (Cases 1-8) were converted from Gram-positive (G(+)) pathogen infection to Gram-negative (G(-)) pathogen infection and two (Cases 9 and 10) were converted from G(-) pathogen to G(+) pathogen infection. Six patients (Cases 11-16) were converted from G(-) pathogen infections to another G(-) pathogen infection. Cases 17-21 had other converted states of implicated pathogens, while Case 18 had a more complicated conversion in different CSF cultures with different combinations of implicated pathogens noted in each culture.

Of the 27 episodes of super-infection, the time interval from finding the previous pathogen to detecting a new pathogen, i.e. appearance of super-infection, ranged from 3 to 90 days. Changes in clinical and laboratory evidence of super-infection were noted in 20 of the 27 episodes. Recurrent fever was the most common and found in 17 episodes (Cases 1-3, 5-8, 10, 11, 14-16, 18, 20), The other new presentations were CSF pleocytosis in 5 episodes (Cases 1, 3, 7, 19, 20), peripheral leukocytosis in 3 episodes (Cases 1, 13, 20), appearance of hydrocephalus in 3 episodes (Cases 2, 14, 19), altered consciousness in 2 episodes (Cases 5, 14), seizure in 1 episode (Case 5), scalp wound infection in 1 episode (Case 18) and appearance of ventriculitis in 1 episode (Case 19). The other 7 episodes without new clinical presentations were detected by regular CSF check-up studies.

Results of antibiotic susceptibilities of the implicated pathogens are listed in Table 2. Initial antibiotic use and the adjusted antibiotic after identification of the pathogens in the super-infections are listed in Table 1. The choices were guided by results of antimicrobial susceptibility tests. In addition to medical treatment, the other 20 patients except Case 8 had undergone removal of EVD or VPS and insertion of a new EVD or VPS.

**Table 2:** Antibiogram of implicated pathogens in the super-infections of the study patients with adult bacterial meningitis (n = 21)

Case	Pathogens	ROC	CAZ	MAX	MEP	CIP	TIG	TMP-SMX	AMP- SBM
1	ESBL-*Escherichia coli*	R	R	R	S		S		
2	*Stenotrophomonas maltophilia*					S		R	
						R		R	
3	*Acinetobacter baumannii*		I	S	S	R			S
			S	S	R	R			S
4	*Enterobacter cloacae*		S		S	S			
	*Pseudomonas aeruginosa*		S	S	S	S			
5	*Enterobacter cloacae*	S	S		S	S			
6	*Acinetobacter baumannii*		S	S	S	S			
			I	S	S	S			
7	*Acinetobacter spp*	I	S	S	S	S			S
8	*Acinetobacter spp*	S	S	S	S	S			S
9	*Pseudomonas aeruginosa*		S	S	S	S			
			R	S	S				
10	*Acinetobacter baumannii*		S	S	S	S			
11A	*Serratia marcescens*	I	R	R	S				
	*Stenotrophomonas maltophilia*					S		S	
11C	*Stenotrophomonas maltophilia*					R		S	
12	*Pseudomonas aeruginosa*		S	S	S	S			
			R	S	S	R			
	ESBL-*Proteus mirabilis*	R	R	S	S	R			
13	*Pseudomonas putida*		S	S	R	S			
	*Acinetobacter spp*	R	R	R	I	R	S		I
14	*Serratia marcescens*	S	S	S	S	S			
	*Acinetobacter baumannii*		R	R	R	R	S		R
15A	*Morganella morganii*	R	S	S	S	S			
	*Proteus mirabilis*	R	R	R	S	R			
15B	Glucose non-fermenting bacilli	R	R	R	R	I			
16	*Stenotrophomonas maltophilia*					S		S	
	*Pseudomonas aeruginosa*		R	R	S	S			
17	*Acinetobacter lwoffii*		S		S	S			
	*Pseudomonas aeruginosa*		S		S	S			
18A	*Escherichia coli*		R	S	S				
18B	*Acinetobacter baumannii*		R	I	S	R			R
18D	*Stenotrophomonas maltophilia*					S			
19	*Escherichia coli*	S	S	S	S				
	*Enterobacter cloacae*	S	S	S	S				R
21	*Enterobacter aerogenes*	I	I	S	S	S			
		R	R	S	S	S			

Abbreviations: ROC, ceftriaxone; CAZ, ceftazidime; MAX, cefepime; MEP, meropenem; CIP, ciprofloxacin; AMP-SBM, ampicillin-subactam; TIG, tigecycline; TMP-SMX, trimethoprime-sulfamethoxazole; LEV, levofloxacin; ESBL, extended spectrum β-lactamase; R, resistant; I, intermediate; S, susceptible

Note: ESBL-producing *Escherichia coli *was suspected by the disk-diffusion susceptibility test [10].

Non-Cephalosporin-susceptible Gram-negative bacteria (GNB) were used to describe isolates that were non-susceptible to ceftriaxone, ceftazidime, and cefepime. Intermediate and resistant isolates were considered non-susceptible [11]. Multi-drugs resistant (MDR) GNB was used to describe isolates that were non-susceptible to all antibiotics routinely tested including amikacin, (ampicillin-sulbactam in *A. baumannii*), ceftriaxone, ceftazidime, cefepime, ciprofloxacin, imepenem, and meropenem [12,13]. The antimicrobial susceptibilities TMP-SMX, ciprofloxacin, levofloxacin, and moxifloaxcin of the *Stenotrophomonas maltophilia *strains isolated from the cerebrospinal fluid were determined concomitantly by the broth dilution method as described in the Clinical and Laboratory Standards (CLSI) guidelines.

With medical treatment, 14 patients survived while 7 died. Four of the 14 survivors (Cases 2, 6, 7, and 15) had clear consciousness while the other 10 had disturbed consciousness. One of the four survivors with clear consciousness (Case 7) had full recovery while the other three had quadriparesis. With GOS assessment, Case 7 had good prognosis, while the other 20 had poor prognosis. Comparative results between the fatal and non-fatal groups showed no significant clinical factors (Table 3).

**Table 3:** Clinical comparison between the fatal and non-fatal groups of the 21 adult bacterial meningitis patients with super-infection

Factors	Non-fatal	Fatal	*P*
	(n = 14)	(n = 7)	
Age, median (range)	48 (25 - 73)	55 (41 - 67)	0.400
Gender			
Male	8	5	0.656
Female	6	2	
Diabetes mellitus			
Yes	1	2	0.247
No	13	5	
Presence of new symptoms			
Yes	10	5	1.000
No	4	2	
Fever			
Yes	10	4	0.638
No	4	3	
Group			
G(+) > G (-) or G(+) > G(+)	7	3	1.000
G(-) → G (-)	5	1	
Intracranial hemorrhage (ICH)			
Spontaneous ICH	4	4	0.085
Traumatic ICH	6	0	

Abbreviation: G(+), Gram-positive pathogen; G(-), Gram-negative pathogen

## Discussion

There is an increasing number of ABM patients with preceding neurosurgical condition, according to previous epidemiologic studies [4,14-16]. A preceding neurosurgical state is associated with mixed, relapsing, or recurrent infectious state of ABM [3,4,8]. In the current study, all 21 ABM patients with super-infection had a neurosurgical condition as the preceding event and all had undergone a procedure involving the insertion of a neurosurgical device during the entire clinical course. This may imply that neurosurgical device insertion is an important event in the development of super-infection. Aside from this pre-condition, 69% (18/26) of the super-infection episodes occurred despite an antibiotic-treatment course for bacterial meningitis longer than two weeks (Table 1). Thus, the occurrence of super-infection during ABM treatment may also suggest an inadequate infectious control program of the hospital.

Due to the preceding neurosurgical condition and existing bacterial meningitis, the clinical presentation of super-infection in ABM may be obscured. In the 27 episodes of the 21 ABM patients in this study, new evidence of meningitis is noted in 74.1% (20/27). Recurrence of fever was the most common, occurring in 63.0% (17/27) of the episodes. Other new evidences of meningeal infection included CSF pleocytosis, peripheral leukocytosis, and altered consciousness. All of these presentations are not unique and can be found in other infectious states, including central nervous system infections [6]. Therefore, a high index of suspicion is important because thus far, only repeat CSF follow-up studies and bacterial culture are the most important steps to confirm the diagnosis of ABM with super-infection.

Staphylococcal species were the most common G(+) pathogens implicated in both initial ABM and ABM with super-infection in the 21 study patients (Table 1). This finding is consistent with previous reports [17-19] that have shown the high prevalence of staphylococcal infection in this specific group of patients. Most of the implicated staphylococcal pathogens shown in this study were methicillin-resistant. However, the presence of these resistant G(+) strains is not a therapeutic problem because intravenous vancomycin is one of the empiric antibiotics for ABM treatment in the hospital [4,16].

It is known that early and appropriate antimicrobial use is of strategic importance in managing ABM [4,5,7]. Early diagnosis of super-infection in the therapeutic course is also important because most patients need an adjustment of antimicrobial agents after identification of the newly-implicated pathogens (Table 1). Such adjustments are important in Cases 1-10 because of the conversion from G(+) to G(-) bacterial pathogens in Cases 1-8 and from G(-) to G(+) pathogens in Cases 9 and 10.

Another important therapeutic consideration in antibiotic use is the emergence of multi-drug resistant bacterial strains in G(-) ABM [11-13,20-25]. This problem is notable especially in patients with underlying G(-) ABM and a super-infection caused by another G(-) pathogen. Case 12 had ESBL-*Proteus mirabilis *infection, Cases 13 and 14 had multi-drug resistant (MDR) *Acinetobacter spp*. infections, and Case 16 had ceftazidime- and cefepime-resistant *Pseudomonas aeruginosa *infection. All four cases needed antibiotic adjustments for the corresponding newly-cultured bacterial pathogens. The emergence of *Stenotrophomonas maltophilia *as the implicated pathogen was noted in Cases 2, 11, and 18. Its emergence has caused a therapeutic challenge because it is usually not susceptible to traditional antibiotics used in ABM treatment [24,25]. Presently, trimethoprime-sulfamethoxazole or quinolones are the antimicrobial agents of choice [23,24].

In this study, another observed problem is the change of antibiotic susceptibilities during the therapeutic course of super-infection by G(-) pathogens (Table 2). MDR *Stenotrophomonas maltophilia*-strain emerged in Case 2 and meropenem-resistant *A. Baumannii *strain in Case 3. Other resistant strains that emerged during the therapeutic course of super-infection were ceftazidime-resistant *A. baumannii *in Case 6, ceftazidime-resistant *Pseudomonas aeruginosa *in Cases 9 and 12, and ceftazidime-resistant *Enterobacter cloacae *in Case 20. All of these changes in antibiotic susceptibilities required an adjustment of antimicrobial agents. In the super-infection of Case 18, anaerobic pathogens appeared and metronidazole was administered thereafter.

The appearance of anaerobic pathogens may also pose a therapeutic challenge because metronidazole is not a regular antimicrobial agent used in ABM treatment [3-7]. Other complicated super-infections in ABM were noted in Cases 11 and 18, in which *Candida spp *was the implicated pathogen. This uncommon super-infection was also noted in the report of Gelfand et al. [2]. Chronic use of broad spectrum antimicrobial agents is an important factor known to encourage the colonization of *Candida *organisms. Antifungal agents should be used for such an uncommon super-infection in treating ABM.

With an effort of management, the therapeutic result of super-infection ABM showed that 95.2% (20/21) of the involved patients belonged to the poor prognosis group. The mortality rate of this specific group of ABM patients is high (33.3%, 7/21) and survivors also have a high incidence of severe morbidity. Because most patients with super-infection ABM have both a preceding neurosurgical event and a pre-existing bacterial meningitis, it is difficult to assess the therapeutic outcome of this uncommon infectious state.

## Conclusions

Super-infection in ABM is usually seen in patients with preceding neurosurgical event, including insertion of an external ventricular drainage device. Fever recurrence is the most common presentation and most new evidences are non-specific. Repeat CSF culture study is needed for diagnostic confirmation. The occurrence of super-infection in ABM has a great impact on the ABM treatment because a high percentage of the implicated bacteria strains are non-susceptible to common antibiotics used and early detection is important for adjusting the antibiotic regimen. Infections by unusual pathogens like anaerobes and fungi may appear and these deserve special attention because the choice of antimicrobials will be different. Because the pre-existing neurosurgical condition is complicated by super-infection, prognosis of super-infection in ABM remains poor. The interpretation of clinical data shown in this study is limited by its small case series and the retrospective data collection in a single hospital-based study. Further large-scale study is needed for better delineation of the presentation and prognosis.

## Competing interests

The authors declare that they have no competing interests.

## Authors' contributions

All authors have read and approved the final manuscript. CRH contributed to the conception and design, data acquisition and analysis, and drafting and revision the manuscript; SFC, CHL, YCC, NWT, CCC, HCW and CCC contributed to the conception and design, and clinical data analysis; and WNC contributed to the conception and design, data analysis, and critical revision and final approval of the revised manuscript.

## Pre-publication history

The pre-publication history for this paper can be accessed here:

http://www.biomedcentral.com/1471-2334/11/133/prepub
